# Leg Lymphoedema After Inguinal and Ilio-Inguinal Lymphadenectomy for Melanoma: Results from a Prospective, Randomised Trial

**DOI:** 10.1245/s10434-024-15149-4

**Published:** 2024-03-17

**Authors:** T. S. Lee, I. Li, B. Peric, R. P. M. Saw, J. P. Duprat, E. Bertolli, J. B. Spillane, B. L. van Leeuwen, M. Moncrieff, A. Sommariva, C. P. Allan, J. H. W. de Wilt, R. Pritchard- Jones, J. L. C. Geh, J. R. Howle, A. J. Spillane

**Affiliations:** 1 Melanoma Institute Australia, Wollstonecraft, Sydney, Australia; 2 Royal North Shore Hospital, Sydney, Australia; 3 University of Sydney, Sydney, Australia; 4 Medical Faculty, Institute of Oncology Ljubljana, University of Ljubljana, Ljubljana, Slovenia; 5 Royal Prince Alfred Hospital, Sydney, Australia; 6 Mater Misericordiae Hospital, North Sydney, Australia; 7 AC Camargo Cancer Center, São Paulo, Brazil; 8 Department of Surgical Oncology, Peter MacCallum Cancer Centre, Melbourne, Australia; 9 Surgical Oncology, University Medical Center Groningen, Groningen, The Netherlands; 10 Norfolk & Norwich University Hospital, Plastic and Reconstructive Surgery, Norwich, UK; 11 Veneto Institute of Oncology IOV-IRCCS, Surgical Oncology, Padua, Italy; 12 Faculty of Medicine, Mater Clinic School, University of Queensland, Brisbane, Australia; 13 Department of Surgery, Radboud University Medical Center, Nijmegen, The Netherlands; 14 Mersey and West Lancashire Teaching Hospitals NHS Trust, Prescot, Knowsley, UK; 15 Department of Plastic and Reconstructive Surgery, St Thomas’ Hospital, Guy’s and St. Thomas’ NHS Foundation Trust, London, UK; 16 Westmead Hospital, Sydney, Australia

## Abstract

**Background:**

The Evaluation of Groin Lymphadenectomy Extent for Melanoma (EAGLE FM) study sought to address the question of whether to perform inguinal (IL) or ilio-inguinal lymphadenectomy (I-IL) for patients with inguinal nodal metastatic melanoma who have no clinical or imaging evidence of pelvic disease. Primary outcome measure was disease-free survival at 5 years, and secondary endpoints included lymphoedema.

**Methods:**

EAGLE FM was designed to recruit 634 patients but closed with 88 patients randomised because of slow recruitment and changes in melanoma management. Lymphoedema assessments occurred preoperatively and at 6, 12, 18, and 24 months postoperatively. Lymphoedema was defined as Inter-Limb Volume Difference (ILVD) > 10%, Lymphoedema Index (L-Dex^®^) > 10 or change of L-Dex^® ^> 10 from baseline.

**Results:**

Prevalence of leg lymphoedema between the two groups was similar but numerically higher for I-IL at all time points in the first 24 months of follow-up; highest at 6 months (45.9% IL [CI 29.9–62.0%], 54.1% I-IL [CI 38.0–70.1%]) and lowest at 18 months (18.8% IL [CI 5.2–32.3%], 41.4% I-IL [CI 23.5–59.3%]). Median ILVD at 24 months for those affected by lymphoedema was 14.5% (IQR 10.6–18.7%) and L-Dex^®^ was 12.6 (IQR 9.0–17.2). There was not enough statistical evidence to support associations between lymphoedema and extent of surgery, radiotherapy, or wound infection.

**Conclusions:**

Despite a trend for patients who had I-IL to have greater lymphoedema prevalence than IL in the first 24 months after surgery, our study’s small sample did not have the statistical evidence to support an overall difference between the surgical groups.

**Supplementary Information:**

The online version contains supplementary material available at 10.1245/s10434-024-15149-4.

The question of whether to perform an inguinal (IL) or ilio-inguinal lymphadenectomy (I-IL) for patients with metastatic melanoma to the inguinal lymph nodes with no clinical or imaging evidence of pelvic disease has been controversial and inconsistently resolved in different institutions for many years.^1^ There is uncertainty about the incidence of pelvic node involvement in patients with metastatic inguinal disease and whether patients benefit from the addition of pelvic lymphadenectomy in terms of a lower rate of disease recurrence, which has been reported in those with node-positive disease.^2^ Any oncological advantage of I-IL over IL needs to be balanced with the disadvantage of the potential extra morbidities related to pelvic dissection.^3–5^ These include wound infections, bleeding, hernia, dehiscence/necrosis, seroma, and lymphoedema.^3^

A meta-analysis of 20 studies in 2016 found that leg lymphoedema developed in approximately 33% (range 25–42%) of patients who underwent IL or I-IL for melanoma.^4^ Chronic physical discomfort and reduced function can plague lymphoedema patients, as well as troublesome recurrent infections and negative body image.^6^ Additionally, lymphoedema can result in significant financial burden on those affected because of treatment and compression garment expenses, time off work, and sometimes, the need to change occupations.^7^

It is generally thought that IL results in lower rates of lymphoedema than I-IL because of fewer removed lymph nodes and less disruption of lymphatic pathways. This view is supported by a previous cross-sectional study.^5^ There are, however, conflicting results arising from other studies. The prospectively designed MSLT^3^ and ANZMTG 01.02/TROG 02.01^8^ trials failed to identify increased lymphoedema risk in those who underwent additional pelvic dissection. A recent Canadian retrospective study also arrived at the same conclusion, but lymphoedema was not objectively measured.^9^ The variability in definition and measurement of lymphoedema, as well as follow-up duration, contributed to inconsistencies in the results.

The Evaluation of Groin Lymphadenectomy Extent for Melanoma (EAGLE FM) study sought to determine if there was a difference in disease-free survival at 5 years after randomisation to IL or I-IL. Secondary endpoints included the prevalence of lymphoedema and quality of life. This EAGLE FM substudy prospectively compared the prevalence and severity of lymphoedema between patients who have had IL and I-IL.

## Methods

The EAGLE FM trial is an international, multicentre, phase III, noninferiority, prospective, randomised clinical trial, which commenced in 2015. The study recruited patients from 14 hospitals across Australia, Brazil, Italy, The Netherlands, Slovenia, and the United Kingdom. Patients were identified through participation of the surgeon and/or other multidisciplinary team members as co-investigators on the clinical trial.

Primary outcome measure was disease-free survival at 5 years after randomisation. Secondary endpoints included the prevalence of lymphoedema and quality of life. The EAGLE FM trial was suspended prematurely in 2021 because of rapid changes in systemic therapy for stage III melanoma and cessation of completion lymph node dissection for positive sentinel node biopsy after the results of MSLT II^10^ and DeCOG SLT.^11^ The lymphoedema substudy was originally intended to follow-up patients to 60 months, but it was discontinued early after 24 months of follow-up because of participant attrition. EAGLE FM follow-up is still ongoing for the primary and recurrence-related secondary outcomes.

Patients were eligible if they had a Stage III inguinal nodal metastatic melanoma from a primary cutaneous melanoma or no known primary tumour with one or multiple inguinal node(s) involved, histologically or cytologically proven as metastatic melanoma, ECOG performance status between 0 and 2 at randomisation, and were able to provide informed consent. Patients were excluded if there was distant metastatic disease on clinical examination or staging imaging, pelvic lymph involvement on sentinel node biopsy or PET/CT scan, and/or bilateral inguinal lymph node involvement. Please refer to supplementary files for full eligibility criteria and surgical protocol. Ethics approval was obtained from each institution’s review board. This trial was registered in the U.S. National Library of Medicine Clinical Trials Registry (NCT02166788).

A minimisation method was applied to randomise patients continuously into IL or I-IL groups using the MinimPy program.^12^ The randomisation was stratified by centre, gender, age, macroscopic versus microscopic lymph node involvement, extracapsular spread versus no extracapsular spread, and primary melanoma (known vs. unknown primary).

### Lymphoedema Substudy

Lymphoedema-related assessments occurred preoperatively at baseline and at 6, 12, 18, and 24 months after surgery. The lymphoedema assessor was blinded to knowledge about the surgical groups.

Lymphoedema-related assessments included the following:

1. Circumference measurement and volume calculations using a measurement board, marking pencil, and tape measure.
2. Bioimpedance spectroscopy where available.
3. Muscle length assessments; Thomas test for hip flexor length and knee-to-wall test for gastrocnemius/soleus length.
4. Lymphoedema Quality of Life questionnaire (LYMQOL) for patients who were identified to have lymphoedema via volumetric difference or bioimpedance.

#### Circumferential Measures and Volume Calculations

Patients were measured in the supine position by using a measurement board placed under each leg. Circumferences were taken by using a tape measure at 10-cm intervals along the affected and unaffected legs.^13,14^ The raw circumferences were converted to limb volumes using a truncated cone formula, and Inter-limb Volume Differences (ILVD) were calculated as a percentage.

#### Multifrequency Bioimpedance Spectroscopy

Bioimpedance spectroscopy utilises the characteristics of frequency-dependent current flow to quantify changes in extracellular fluid in a participant’s limb. Impedimed SFB7 and U400 were used to perform bioimpedance spectroscopy. Both machines measure the resistance to current flow in the extracellular fluid of the operated leg compared to the contralateral side via use of surface skin electrodes, producing a Lymphoedema Index (L-Dex^®^). Bioimpedance spectroscopy is unaffected by variations in the participant’s weight due to a change in fat mass or muscle mass and provides an instant tool for assisting in the clinical assessment of lymphoedema change.

Lymphoedema thresholds were defined based on the International Lymphoedema Framework Best Practice Consensus Report and the few published clinical trials available for lower limb lymphoedema at that time of study inception.^5,15–17^

1. ILVD >10% compared to the unaffected limb or
2. L-Dex^®^ >10 or
3. L-Dex^®^ change of >10 points compared with baseline.

Participants were referred for lymphoedema management if they had protocol-defined lymphoedema. Lymphoedema management was at the discretion of the treating institution, including the use of compression garments for treatment or prophylaxis. Use of compression garments was recorded at each follow-up and may have started or ceased at, or between, any timepoint/s.

#### Muscle Length Assessments

Hip flexor length was assessed by using the position required for Thomas test. Thomas test is performed with the patient in supine lying with lower back flat on plinth. The contralateral leg is passively flexed to end of range. A positive Thomas test occurs when ipsilateral leg raises off the plinth, and this indicates short hip flexor length. Hip flexor length tests were discontinued once a negative test was achieved. Calf muscle length was measured with the patient standing in a lunge position facing the wall and with affected knee and hip extended and heel flat on the floor. The distance from the wall to the distal first phalanx was measured in this position.

#### LYMQOL

The LYMQOL-LEG questionnaire is a validated condition-specific Quality of Life assessment tool, which can be used for lymphoedema of the limbs both in clinical assessment and as an outcome measure.^18^ This tool contains domains including physical, emotional, social, role functioning, and other symptoms, such as pain and fatigue. Only participants who present with a protocol-defined lymphoedema completed the LYMQOL-LEG questionnaire.

#### Statistical Methods

Sample size was calculated for the hypothesis of the primary study, which is noninferior disease-free survival in IL compared with I-IL, within a margin of 7%, at 5 years after surgery. The purpose was to recruit a sample size of 634 subjects (317 in each group) to achieve 80% power at a 0.05 significance level.

Data were stratified based on the type of lymphadenectomy and the presence or absence of lymphoedema. The relationships between each stratum and independent demographic and treatment factors were analysed by using Fisher’s exact test and Wilcoxon rank-sum test. Linear hypothesis tests were conducted to see if the two surgical groups and their interaction with other variables were associated with different likelihoods of developing lymphoedema at each timepoint. Additionally, a marginal model known as Generalised Estimating Equations (GEE) was employed to statistically quantify the relationship between lymphoedema and these factors, taking into consideration the repeated measurement correlations. Independent variables with a *p*-value < 0.2 and the type of lymphadenectomy were considered in the GEE model. To obtain a more parsimonious model and improve accuracy, insignificant variables were removed from the final multivariate model. The reference group in the model consisted of female IL patients, age < 55 years with nondominant limb affected, average body mass index (BMI) and total node removed, and no compression garment usage.

## Results

**Fig. 1:**
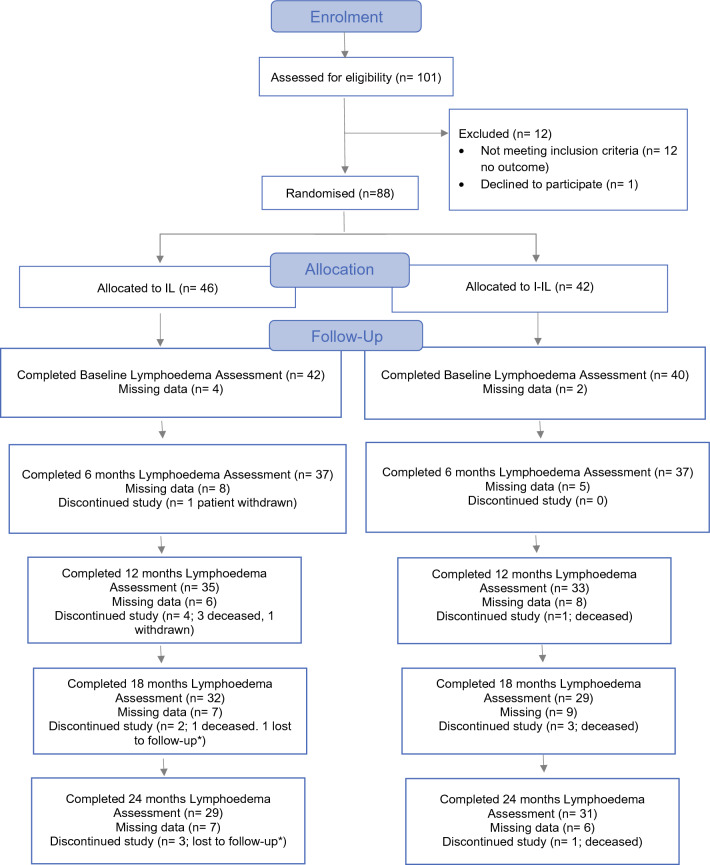
CONSORT diagram. *Lost to follow-up is defined as those who did not attend EAGLE FM study measurements from 24 to 36 months.

Of the 88 patients randomised, 82 completed the lymphoedema assessment at baseline (IL = 42, I-IL = 40), and 60 patients (IL = 29, I-IL = 31) completed it at 24 months (Fig. 1). Reasons for not completing the assessment include missing data and discontinuation of the study due to death and lost to follow-up. Hip flexor and calf length measures were not included in the results due to measurement error for some sites resulting in noninterpretable data. LYMQOL data were not analysed due to large amounts of missing data for the low number of patients with protocol-defined lymphoedema.

At baseline, there were no significant differences between IL and I-IL in terms of gender, age, BMI, dominant limb affected, geographic location, ILVD, or L-Dex^®^ (Table 1). Race and ethnicity data were missing for most sites, because this information was not requested as a compulsory field in the 2015 study protocol. Mean (standard deviation [SD]) number of total lymph nodes was 10.9 (5.1) for IL and 21.5 (7.8) for I-IL patients. There was no significant difference found between the two surgical groups in the number of positive nodes, groin radiotherapy, micrometastases and macrometastases, or wound infection postoperatively. There were 26 serious adverse events (IL = 12, I-IL = 14). These included wound infections, excessive wound drainage, thromboembolism, and syncope. All but one event occurred within 120 days of surgery.

**Table 1:** Patient demographics at baseline

		Total (*N* = 82)	IL (*N* = 42)	I-IL (*N* = 40)	*p*
Gender	Male	36 (43.9%)	19 (45.2%)	17 (42.5%)	0.827
	Female	46 (56.1%)	23 (54.8%)	23 (57.5%)	
Age (year)	Mean (SD)	56 (12)	56 (12)	56 (13)	0.853
Body mass index	Mean (SD)	26.5 (5.3)	26.9 (4.2)	26.1 (6.2)	0.854
Dominant limb affected	Yes	38 (46.3%)	18 (42.9%)	20 (50.0%)	0.658
	No	44 (53.7%)	24 (57.1%)	20 (50.0%)	
Geographic location	Australia	35 (42.7%)	17 (40.5%)	18 (45.0%)	0.907
	South America	9 (11.0%)	4 (9.5%)	5 (12.5%)	
	Europe	38 (46.3%)	21 (50.0%)	17 (42.5%)	
Lymphoedema	Yes	6 (7.3%)	5 (11.9%)	1 (2.5%)	0.202
	No	76 (92.7%)	37 (88.1%)	39 (97.5%)	
ILVD	Median (IQR)	0.9 (− 1.4, 3.9)	1.8 (− 1.8, 5.5)	0.5 (− 1.1, 2.6)	0.303
L-Dex^®^	Median (IQR)	2.8 (− 1.6, 4.7)	0.7 (− 2.2, 3.3)	4.0 (1.4, 5.2)	0.075

Baseline L-Dex^®^ was captured for 37 participants (45%), but this reduced to 24 participants (40%) by 24 months. All available participants underwent circumferential measurement for limb volume throughout the study. Some patients with leg lymphoedema were detected by using the ILVD >10% definition but were not captured by L-Dex^® ^> 10, whereas some were detected by L-Dex^®^ and not by ILVD (Table 2).

**Table 2:** Lymphoedema detection methods

Time	ILVD *N* (%)	L-Dex^®^ *N* (%)	ILVD and L-Dex^®^ (joint detection) *N* (%)
Baseline	3 (50.0%)	3 (50.0%)	0 (0.0%)
6	31 (83.8%)	3 (8.1%)	3 (8.1%)
12	15 (60.0%)	5 (20.0%)	5 (20.0%)
18	13 (72.2%)	1 (5.6%)	4 (22.2%)
24	13 (56.5%)	5 (21.7%)	5 (21.7%)

**Fig. 2:**
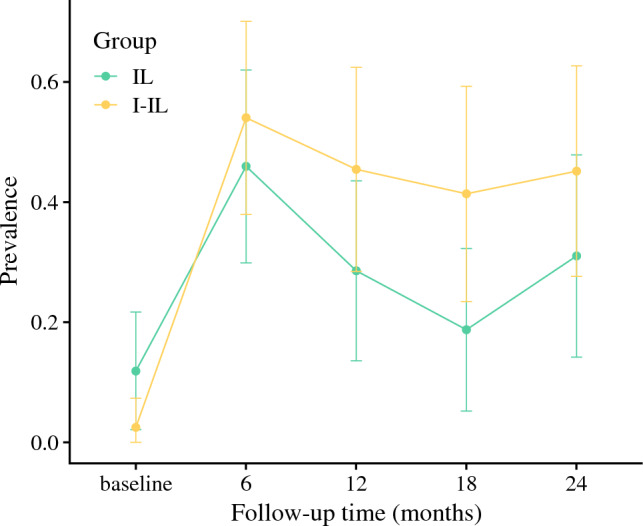
Lymphoedema prevalence over time

At baseline, the prevalence of leg lymphoedema was higher for IL (5/42) compared with I-IL (1/40). Peak lymphoedema prevalence occurred at 6 months for both lymphadenectomy groups, with approximately one in every two patients affected (45.9% IL [CI 29.9–62.0%], 54.1% I-IL [CI 38.0–70.1%]). Table 3 presents the summary of variables stratified by lymphoedema at 6 months. Lymphoedema prevalence decreased from 6 months, affecting 18.8% [CI 5.2–32.3%] of IL and 41.4% [CI 23.5–59.3%] of I-IL patients at 18 months. Between 18 and 24 months, the prevalence of lymphoedema rose for both surgical groups but the prevalence remained lower than at 6 months (IL 31.0% [CI 14.2–47.9%]; I-IL 45.2% [CI 27.6–62.7%]). Although there is no statistical difference between the surgical groups overall, I-IL patients had a higher lymphoedema prevalence than IL patients during all follow-up timepoints (Fig. 2).

**Table 3:** Summary of variables stratified by lymphoedema at 6 months

		Total (*N* = 74)	No lymphoedema (*N* = 37)	Lymphoedema (*N* = 37)	*p*
Age (year)	Mean (SD)	56 (12)	56 (12)	56 (13)	0.853
Body mass index	Mean (SD)	26.5 (5.3)	26.9 (4.2)	26.1 (6.2)	0.854
Dominant limb affected	Yes	38 (46.3%)	18 (42.9%)	20 (50.0%)	0.658
	No	44 (53.7%)	24 (57.1%)	20 (50.0%)	
Lymphadenectomy group	IL	37 (50.0%)	20 (54.1%)	17 (45.9%)	0.642
	I-IL	37 (50.0%)	17 (45.9%)	20 (54.1%)	
Number of removed nodes*	Mean (SD)	17.3 (8.6)	16.5 (8.1)	18.1 (9.0)	0.596
Number of positive nodes*	Mean (SD)	1.8 (1.6)	1.5 (1.1)	2.0 (2.0)	0.227
Metastatic foci	Micro	34 (45.9%)	16 (43.2%)	18 (48.6%)	0.816
	Macro	40 (54.1%)	21 (56.8%)	19 (51.4%)	
Primary excision site	Foot	9 (12.2%)	4 (10.8%)	5 (13.5%)	0.132
	Leg	44 (59.5%)	18 (48.6%)	26 (70.3%)	
	Trunk	12 (16.2%)	8 (21.6%)	4 (10.8%)	
	Missing	9 (12.2%)	7 (18.9%)	2 (5.4%)	
Local or in-transit recurrence	Yes	19 (25.7%)	8 (21.6%)	11 (29.7%)	0.604
	No	28 (37.8%)	16 (43.2%)	12 (32.4%)	
	Missing	27 (36.5%)	13 (35.1%)	14 (37.8%)	
Regional recurrence	Yes	14 (18.9%)	6 (16.2%)	8 (21.6%)	0.825
	No	33 (44.6%)	18 (48.6%)	15 (40.5%)	
	Missing	27 (36.5%)	13 (35.1%)	14 (37.8%)	
Wound infection	Yes	19 (25.7%)	10 (27.0%)	9 (24.3%)	0.999
	No	55 (74.3%)	27 (73.0%)	28 (75.7%)	
Radiotherapy (groin)	Yes	15 (20.3%)	7 (18.9%)	8 (21.6%)	0.999
	No	59 (79.7%)	30 (81.1%)	29 (78.4%)	
Compression garment	Yes	39 (52.7%)	21 (56.8%)	18 (48.6%)	0.642
	No	35 (47.3%)	16 (43.2%)	19 (51.4%)	

*Number of removed and positive nodes include nodes removed at initial sentinel node biopsy

**Fig. 3:**
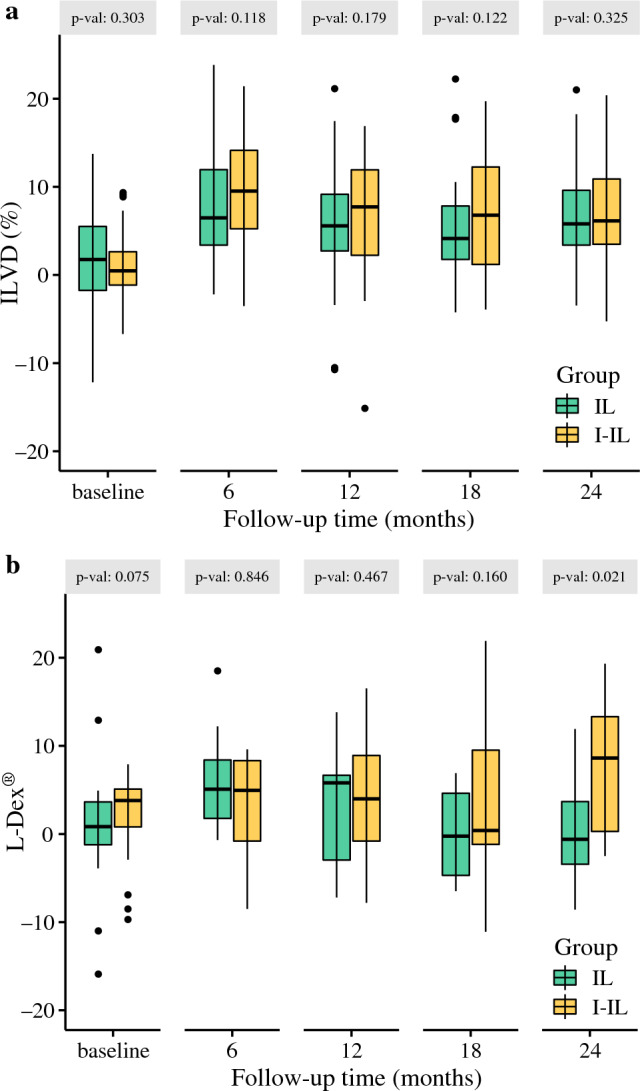
**a** Interlimb volume difference over time. **b** L-Dex^®^ over time

At 6 months, median ILVD was 6.5% (IQR 3.4–11.9%) and 10.5% (IQR 5.6–14.7%) and for those measured with bioimpedance, median L-Dex® was 5.1 (IQR 1.8–8.4) and 5.1 (IQR − 0.3 to 8.7) for IL and I-IL respectively. At 24 months, median ILVD remained low for both groups at 5.8% (IQR 3.4–9.6%) and 7.2% (IQR 3.6–14.0%) for IL and I-IL, but median L-Dex^®^ was significantly higher for I-IL at 9.1 (IQR 0.9–13.3) compared with − 0.6 (IQR − 3.4 to 3.7) for IL (Fig. 3a, b).

The severity of leg lymphoedema was mild in most cases detected at 6 months, with a median ILVD of 13.8% (IQR 11.2–17.3%) and L-Dex^®^ of 9.4 (IQR 7.0–11.4). At 24 months, lymphoedema severity increased slightly for those affected, with median ILVD at 14.5% (IQR 10.6–18.7%) and median L-Dex^®^ at 12.6 (IQR 9.0–17.2).

Recurrence data were available for 59% (*n *= 48) of our participants. Recurrence did not differ significantly between IL and I-IL groups, nor was it associated with the development of lymphoedema during follow-up. Local or in-transit recurrence occurred in 21.4% of IL and 25% of I-IL (*p *= 0.955) and regional recurrence occurred in 26.2% of IL and 15% of I-IL (*p *= 0.334).

Interestingly, 52.7% of all patients (*n *= 74) were using compression garments at 6 months, irrespective of whether they had lymphoedema. By 24 months, compression garment use had decreased to 41.7% (*n *= 60); only ten (43.5%) of the 23 patients with lymphoedema used compression. The pattern of compression garment use appeared to follow the lymphoedema prevalence of the lymphadenectomy group at that timepoint; higher usage corresponded to higher prevalence. For example, in the IL group, highest lymphoedema prevalence at 6 months of 45.9% corresponded with highest compression garment use in 43.2% of patients, whereas lowest lymphoedema prevalence at 18 months of 18.8% corresponded with lowest compression garment use in 28.1% of patients.

**Fig. 4:**
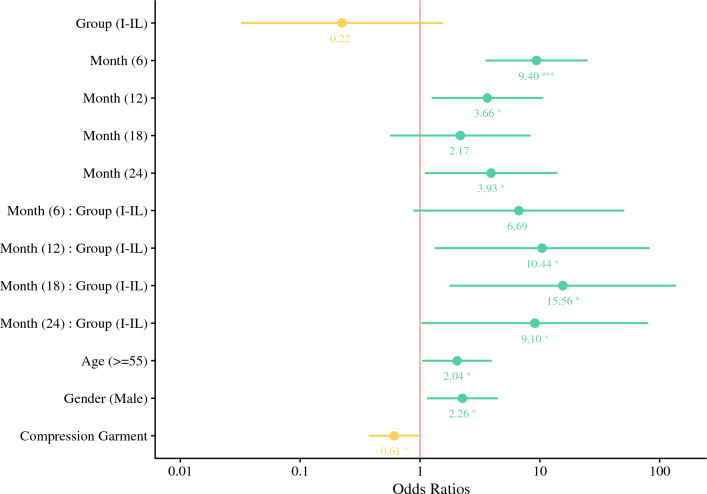
Odds ratios for lymphoedema

The final fitted multivariate model showed that the odds of developing lymphoedema between the IL and I-IL groups depends on time (Fig. 4). The odds ratio (OR) between the surgical groups substantially increased from the baseline, reaching around 10 (OR = 10.44; CI 1.32–82.40; *p* = 0.026), 16 (OR = 15.56; CI 1.77–137.18; *p* = 0.013), and 9 (OR = 9.10; CI 1.03–80.38; *p* = 0.047) times higher at 12, 18, and 24 months, respectively. Additionally, the likelihood of developing lymphoedema reached its highest point at 6 months (OR = 9.40; CI 3.53–25.04; *p* < 0.001). Linear hypothesis testing demonstrated a significant difference between I-IL and IL in their chance of developing lymphoedema at 18 months (OR = 3.49; CI = 1.15–10.56; *p* = 0.027). However, there was no statistical evidence to support a difference at any other timepoint or overall difference (baseline: OR = 0.22; CI 0.03–1.56; *p* = 0.131, 6 m: OR = 1.5; CI 0.58–3.85; *p* = 0.399, 12 m: OR = 2.34; CI 0.82–6.68; *p* = 0.113, 24 m: OR = 2.04; CI 0.69–5.99; *p* = 0.195, overall: *p* = 0.092).

Our multivariate model also indicated that patients older than 55 years were twice as likely to be affected by lymphoedema than those younger than 55 years (OR = 2.04; CI 1.04–4.00; *p* = 0.038). Male patients were associated with a significantly higher risk of lymphoedema development compared with female patients (OR = 2.26; CI 1.14–4.47; *p* = 0.019). Patients who used compression garments had a significantly reduced prevalence of lymphoedema by 39% on average (OR = 0.61; CI 0.37–1.00, *p* = 0.049).

## Discussion

Despite slow recruitment and smaller than anticipated participant numbers, EAGLE FM remains the largest, prospective, randomised, controlled trial addressing the extent of surgery for inguinal nodal metastatic melanoma. Access to modern adjuvant therapies in the management of melanoma is unequal worldwide. For many patients, lymphadenectomy is still necessary for disease control. Additionally, there are patients whose comorbidities prevent them from receiving immunotherapy or targeted therapy, as well as nonresponders to these treatments.

Because of lack of statistical evidence, we cannot report whether there is any difference in lymphoedema prevalence between the surgical groups in general over the course of follow-up. However, I-IL patients did show a significantly higher chance of developing lymphoedema at 18 months compared with IL. This is consistent with the results of our previous study that indicate a higher overall prevalence for patients who have I-IL versus IL.^5^

Our multivariate model also confirms the odds ratio of lymphoedema between I-IL and IL increased substantially at 12, 18 and 24 months follow-up. It is possible that the effects of the more extensive I-IL are compounded over time so that factors, such as deposition of scar tissue blocking lymph pathways and lymphangiogenesis, and chronic tissue inflammation, do not become apparent until 12 months or later from surgery.^19,20^

The prevalence of lymphoedema was similarly high for both IL and I-IL patients at 6 months and declined for both groups from 6 to 18 months but increased again at 24 months. This would imply that for some patients, leg lymphoedema detected at 6 months, is reducible and may be reversible. It is unclear whether it is natural recovery over time or lymphoedema treatment that helped reduced the leg oedema in these patients. Regardless, this may help to reassure patients that not everyone who has leg oedema at 6 months develop chronic lymphoedema.

The severity of lymphoedema for IL and I-IL was mostly mild during follow-up. The fact that our participants were monitored regularly, similar to a prospective surveillance model, may have contributed to cases being detected early and referrals made early for lymphoedema treatment, thereby reducing the overall severity and prevalence. It is well-known that a prospective surveillance model of care for breast cancer patients reduces the prevalence and burden of chronic lymphoedema and the cost to the health system.^21,22^ This model of care can be generalised to benefit melanoma patients who have had lymphadenectomies and the health system that treats them.

Leg lymphoedema detected by ILVD>10% did not always correspond with an L-Dex^® ^> 10. Since developing the EAGLE FM protocol in 2015, there has been a lowering of the lymphoedema threshold from 10 to 7, which is reflective of a change from 3 to 2SD away from the average estimate of the healthy population.^23^We reanalysed the data with the new threshold of 7, and it improved the joint detection rate of ILVD and L-Dex^®^ by four patients at 6 months and one patient at 18 months, but these patients were already identified to have lymphoedema by the ILVD definition in our original analysis. Ultimately, lowering the threshold did not change the overall lymphoedema prevalence in this study. Objective lymphoedema definitions remain contentious; this extends to the ILVD threshold, with calls to both lower and raise the threshold in recent years.^24,25^ Until there is agreement on a universal definition, replication and comparison of studies remain problematic.

Our study demonstrated that older age was associated with an increased risk of lymphoedema. Older age is associated with enlarged lymph vessels and increased permeability in both animal and human models.^26^ Additionally, comorbidities, such as venous insufficiency, is more likely to affect the older age group.^27^ Venous insufficiency may be subclinical before surgery, but the increase in interstitial fluid after surgery can place more demand on fluid return, resulting in both venous and lymphatic failure, and leg lymphoedema.

Around half of all participants were using compression garments at 6 months, irrespective of lymphoedema status. It is unclear which patients were using their compression garments prophylactically and which patients were using their compression garment as treatment for their lymphoedema. This is indeed a limitation of our study. Compression garment use would need to be controlled and consistent during follow-up to evaluate its effectiveness. The pattern of compression garment use in our results for both groups appear to follow the prevalence of lymphoedema for that group at the timepoint of follow-up, which would suggest that they were being used for treatment rather than prophylaxis. Prophylactic use of compression garments has not been proven to reduce lymphoedema risk in melanoma patients after groin dissection, but it has been shown to reduce lymphoedema in high-risk breast cancer patients.^28,29^ Our multivariate model would appear to agree with the latter as our patients who wore compression garment were far less likely to have lymphoedema than those who did not wear compression.

Patients that were using compression garments as treatment for their lymphoedema would be expected to have minimal lymphoedema, potentially underestimating the overall extent of lymphoedema in the study population. On the other hand, it also would appear that many of our patients with lymphoedema did not wear, or adhere, to compression garments despite referral to a lymphoedema clinic. A previous study also found that females were more likely than males to wear compression stockings for the management of venous oedema, which would explain the higher odds of lymphoedema for males in our study.^30^ It is likely that patient perception of threat and their coping strategies, including their perception of lymphoedema being a problem, their ability to don garments, garment cost, comfort, and employment contributes to their adherence to compression.^31–33^

## Conclusions

Despite the trend for higher lymphoedema prevalence in patients who have had I-IL over IL, our study’s small sample did not have the statistical evidence to support an overall difference in lymphoedema prevalence between the surgical groups during 24 months of follow-up. Lymphoedema severity was mostly mild in those affected.

### Supplementary Information

Below is the link to the electronic supplementary material.

Supplementary file1 (DOCX 18 KB)

Supplementary file2 (DOCX 125 KB)

Supplementary file3 (DOC 217 KB)
