# Supplementary material for: Leg Lymphoedema After Inguinal and Ilio-Inguinal Lymphadenectomy for Melanoma: Results from a Prospective, Randomised Trial
Source: Ann Surg Oncol. 2024 Mar 17;31(6):4061–70. doi: 10.1245/s10434-024-15149-4 (PMC11076360; doi:10.1245/s10434-024-15149-4)
Supplement: Supplementary file 1 — Supplementary file1 (DOCX 18 KB) [file 10434_2024_15149_MOESM1_ESM.docx]

**Evaluation of leg lymphoedema after inguinal and ilio-inguinal lymphadenectomy for melanoma. Results from a prospective randomised trial.**

**Study Eligibility Criteria**

(EAGLE FM Trial -PROTOCOL - MASC Trials 01.12 EAGLE FM Protocol version 2.2 dated 14 June 19) <https://www.masc.org.au/active-trials-closed-for-recruitment/>

***Patient population***

Patients will be recruited from participating multi-disciplinary melanoma centres. Patients will be identified through participation of surgeon and / or other multidisciplinary team members as co-investigators in the clinical trial.

***Inclusion criteria***

Patients may be included in the study if they meet **ALL** of the following criteria:

1. Must be 15 and above.
2. Have primary cutaneous melanoma or if the patient presents with stage III melanoma with no known primary tumour then a thorough search for the primary should be documented (including perineal and perianal areas).
3. Life expectancy of at least 10 years from the time of diagnosis, not considering the melanoma in question, as determined by the PI.
4. Must have one or multiple inguinal node(s) involved, histologically or cytologically proven as metastatic melanoma. This can be detected:

- At the time of diagnosis;
- Or by Ultrasound detection;
- Or later after relapse when no Sentinel Node Biopsy (SNB) was performed at the time of primary tumour management;
- Or as a result of SNB;
- Or at the time of regional recurrence after “false negative” SNB;

1. Absent distant disease clinically and on PET/CT scan. (Patients must have NO further distant disease or visceral metastases).
2. ECOG performance status must be between 0 to 2 at randomisation.
3. Whole body PET/CT scan, specifically stating there is NO evidence of pelvic lymph node involvement prior to randomisation and a CT Brain or MRI Brain scan. Scans must be performed within 6 weeks prior to randomisation.
4. Able to provide written, informed consent.
5. Willing to return to the centre for follow up examinations and procedures, as outlined in the protocol.
6. All patients must be randomised and undergo lymphadenectomy surgery no more than 120 days following diagnosis of inguinal LN involvement.

***Exclusion criteria***

Patients will be excluded from the study for **ANY** of the following reasons:

1. Distant metastatic disease on clinical examination or staging imaging (whole body PET/CT scan and CT or MRI brain scan). Scans must be performed within 6 weeks prior to randomisation.
2. Pelvic LN involvement on SNB or PET/CT scan suggestive of metastatic disease in the pelvis – criteria for diagnosis include normal size or enlarged lymph nodes (> 1 cm) with increased FDG activity on PET (SUV >3). If there are enlarged, necrotic lymph nodes FDG activity on PET is not required to be present. If unsure central review facilitated by the Trial Coordinating Centre should be sought.
3. Bilateral inguinal lymph node involvement.
4. Patients with a history of major pelvic surgery and / or regional radiotherapy at any time in the past.
5. Requiring planned radiotherapy following surgery due to macroscopic, bulky and matted nodes.
6. Unfit for General Anaesthesia.
7. Melanoma-related operative procedures not corresponding to criteria described in the protocol.
8. Patients with prior cancers, except:

- those with a thin <=1 mm, regionally unrelated melanoma > 5 years ago.
- those with a good prognosis regionally unrelated cancer (>90% probability of 10 years disease specific survival).
- other cancers diagnosed more than five years ago with no evidence of disease recurrence within this time.
- successfully treated basal cell and squamous cell skin carcinoma.
- carcinoma in-situ of the cervix.
- 1 episode of in transit melanoma > 3 years ago

1. A medical or psychiatric condition that compromises ability to give informed consent or complete the protocol.
2. Positive urine pregnancy test for women of childbearing potential (+/-7 days of randomisation onto the trial).

The patient treatment history (including adjuvant systemic therapy clinical trial participation) will be recorded at the first study visit and as appropriate throughout the duration of the study. Patients can receive treatment using neoadjuvant therapies prior to and during this trial.
