# Supplementary material for: Leg Lymphoedema After Inguinal and Ilio-Inguinal Lymphadenectomy for Melanoma: Results from a Prospective, Randomised Trial
Source: Ann Surg Oncol. 2024 Mar 17;31(6):4061–70. doi: 10.1245/s10434-024-15149-4 (PMC11076360; doi:10.1245/s10434-024-15149-4)
Supplement: Supplementary file 2 — Supplementary file2 (DOCX 125 KB) [file 10434_2024_15149_MOESM2_ESM.docx]

**Evaluation of leg lymphoedema after inguinal and ilio-inguinal lymphadenectomy for melanoma. Results from a prospective randomised trial.**

**Surgical Protocol** (EAGLE FM Trial -PROTOCOL - MASC Trials 01.12 EAGLE FM Protocol version 2.2 dated 14 June 19) <https://www.masc.org.au/active-trials-closed-for-recruitment/>

**Surgical Treatment of Inguinal Disease**

All patients must be randomised and undergo lymphadenectomy surgery no more than 120 days following diagnosis of inguinal LN involvement.

**Inguinal Lymphadenectomy or Ilio-inguinal Lymphadenectomy**

***Surgical Technique***

Standard surgical incisions are eligible. These include long single incisions (straight or curvilinear or sigmoid), separate minimal access incisions sited above and below the inguinal ligament or videoscopic incisions. Saphenous vein sparing is permissible unless there is macroscopic inguinal nodal disease.

Standard thin skin flaps are raised at least 5 centimetres superiorly above the inguinal ligament, laterally to the line of the anterior superior iliac spine and medially to the line of the pubic tubercle (see surface anatomy in **Figure 2**).

**Figure 2**

The dotted line indicates the surface markings of the area resected in an inguinal lymphadenectomy. Minimal access incisions are marked above and below the inguinal ligament marking, but other incision types are acceptable.

Superiorly the fatty tissue is then incised at these limits and deepened onto the abdominal wall. The fatty flap is mobilised inferiorly exposing the external oblique aponeurosis down to the inguinal ligament. The abdominal wall is split or divided in layers in the line of the external oblique, internal oblique and transversus abdominis muscle fibres to gain access to the extra-peritoneal plane. The extra-peritoneal plane is developed inferiorly and then medially along the posterior abdominal wall by blunt finger dissection to expose the common and external iliac vessels. The ureter is swept off these vessels, and the standard iliac node clearance is usually commenced superiorly within 5cm of at the level of the aortic bifurcation on the left, or, within 5 cm of at the level of the lower end of the inferior vena cava on the right. Nodal tissue around the common iliac vessels is resected in-continuity with the external iliac nodes. Particular care is taken to include all the nodal tissue at the lower end along the deep aspect of the inguinal ligament. The obturator component of the dissection is taken as a separate specimen on most occasions. The obturator node dissection is commenced at the superior aspect of the femoral canal and after mobilising that tissue it is dissected further craniad posterior to the external iliac vein, which may have several tributaries requiring division. The depth of dissection into the pelvis is usually back to the obturator nerve and related internal iliac vessels but other nodal tissue is resected if present adjacent to these structures. After confirming adequate clearance of LNs the muscle layers of the abdominal wall are closed with 2 layers of nylon sutures.

In the inguinal area, thin skin flaps are similarly raised for the extent of the femoral triangle. This tissue is mobilised down in the line from anterior superior iliac spine along the lateral edge of the sartorius muscle and as far medial as the tendon of the adductor longus muscle superiorly. The standard IL is then performed inferiorly to the apex of the femoral triangle. The femoral canal is explored separately to ensure continuity with the pelvic dissection. The femoral canal is then repaired with nylon suture. The sartorius muscle may be reflected if the wound has the femoral vessels in the base. Two large suction drains are commonly used. One may be inserted into the cavity laterally and extending to the upper limit of the cavity and one below the dissection extending up to the level of the inguinal ligament. Deep tissues are closed with absorbable sutures and skin is closed with staples or sutures.
